# Personalized Nutrition, Lifestyle, and Supplementation Strategies to Support Cognitive Performance and Well-Being in Esports Athletes: A Narrative Review

**DOI:** 10.3390/nu18060981

**Published:** 2026-03-19

**Authors:** Loizos Georgiou, Irene P. Tzanetakou, Konstantinos Giannakou, André Baumann, Elena Hadjimbei

**Affiliations:** 1 Department of Life Sciences, School of Sciences, European University Cyprus, Nicosia 2404, Cyprus; lg212248@students.euc.ac.cy (L.G.);; 2 Department of Health Sciences, School of Sciences, European University Cyprus, Nicosia 2404, Cyprus; k.giannakou@euc.ac.cy; 3 Gamers Performance, 1185 Oslo, Norway

**Keywords:** esports, athlete performance, nutrition, lifestyle behaviors, dietary habits, cognitive performance, dietary supplements

## Abstract

Esports are a rapidly expanding form of competitive activity that demand high levels of cognitive alertness, motor precision, stress management, and resilience to mental and physical fatigue. At the same time, the sedentary lifestyle, extended screen exposure, and psychological pressures associated with competitive gaming raise concerns for both performance and long-term health. Growing evidence highlights the importance of nutrition and lifestyle behaviors in supporting cognitive performance and overall competitive demands. While balanced dietary patterns and adequate hydration are essential, dietary supplements may provide additional benefits when used appropriately and under professional guidance. However, the current research is limited by a predominance of cross-sectional and self-reported studies, short-term or acute interventions, small sample sizes, and insufficient emphasis on esports-specific and personalized strategies. This review examines existing evidence on individualized nutrition, supplementation, and lifestyle strategies in esports, identifies key methodological limitations, and outlines future directions to inform evidence-based practice for athletes, practitioners, and organizations seeking to optimize cognitive performance, well-being, and long-term sustainability in this emerging field.

## 1. Introduction

Esports, or competitive video gaming, have evolved from a leisure activity into a structured and professionalized part of the global sports and entertainment industry. The global esports market is projected to reach approximately USD 4.5 billion by 2026, and audiences are forecasted to continue growing toward around 900 million users in the coming years, indicating its widening global reach [1,2]. This expanding cultural and economic influence is further reflected by high-profile institutional recognition. In January 2026, six-time League of Legends world champion Lee “Faker” Sang-hyeok was awarded the Cheongyeong Medal by the President of South Korea, marking the first time an esports athlete has received the country’s highest sporting honor and symbolizing broader acceptance of esports within national sports cultures [3]. At the international level, the International Olympic Committee has explored integrating esports into the Olympic Movement, including announcing plans for an “Olympic Esports Games” in partnership with the Saudi Olympic and Paralympic Committee. While a 2027 event in Riyadh was initially discussed, the collaboration was later discontinued, and the IOC stated that it intends to pursue a revised approach without a confirmed host or date at this time [4]. Together, these developments highlight how esports now rival certain traditional sports in both global influence and institutional validation.

Despite being virtual in nature, esports impose substantial demands on athletes. Success in competition requires sustained cognitive alertness, precise motor coordination, rapid decision-making under pressure, and resilience to mental and physical fatigue [5,6]. Concurrently, the lifestyle associated with esports, characterized by physical inactivity, extended screen time, disrupted sleep cycles, and high psychological stress, poses risks to both short and long-term health [7,8]. These challenges highlight the need for tailored strategies that not only optimize in-game performance but also safeguard athlete well-being, including adequate sleep and recovery practices [5,6,7,8].

Emerging evidence suggests that nutrition, dietary supplementation, and lifestyle habits play a crucial role in shaping both cognitive and physical performance in esports athletes [7]. Balanced dietary patterns, adequate hydration, and sufficient sleep duration and quality are consistently linked to improved mental clarity, reaction times, and stress management [7,8]. Similarly, targeted use of supplements and structured physical activity has been proposed as means to counteract fatigue, musculoskeletal strain, and mental burnout [9,10,11]. However, most existing studies remain limited in scope, relying on self-reports, short-term interventions, or non-standardized measures, while personalized approaches remain scarce [7,9,10,11,12].

Specifically, much of the existing literature frequently borrows frameworks from traditional sports without esports-specific validation, prioritizes acute supplementation outcomes over long-term adaptations, and rarely considers individualized nutrition or interactions with broader lifestyle factors such as sleep, stress, and physical activity.

This review seeks to address these gaps by examining the significance of individualized nutrition, supplementation, and lifestyle practices in esports. By consolidating recent findings, it aims to provide an overview of existing knowledge, highlight key methodological limitations, and propose directions for future research. Furthermore, it evaluates the emerging role of digital and AI-driven tools for individualized monitoring and intervention, while addressing the ethical and practical risks associated with their implementation in esports. In doing so, it underlines the importance of approaching esports not merely as a form of digital entertainment but as a demanding competitive field that requires the same level of scientific attention as traditional sports.

## 2. Review Design and Literature Search Strategy

This narrative review was informed by a structured literature search conducted across PubMed, Scopus, and Web of Science. The search covered publications from 2005 to 2025 and combined terms related to “esports” or “competitive gaming” with “nutrition,” “dietary supplements,” “cognition,” “lifestyle behaviors,” “physical activity,” “sleep,” and “performance.”

Eligible studies included peer-reviewed publications in English involving human participants engaged in esports or competitive gaming. The population of interest comprised adolescent and adult esports players, competitive gamers, and elite esports athletes. Both professional and amateur competitors were considered.

A range of study designs was included, reflecting the emerging nature of the field. These comprised cross-sectional studies, cohort studies, randomized controlled trials, experimental laboratory studies, intervention studies, and relevant systematic reviews. Where esports-specific evidence was limited, mechanistic or comparative studies conducted in traditional athletic or cognitive-performance populations were included to contextualize findings and provide mechanistic insight. These studies were considered to inform potential physiological and cognitive mechanisms relevant to esports performance, while acknowledging that direct applicability to competitive gaming environments remains to be fully established.

Exclusion criteria included studies focusing exclusively on non-competitive gaming, pediatric or clinical populations unrelated to esports performance, non-human studies, conference abstracts, commentaries, and non-peer-reviewed sources.

After screening titles, abstracts, and full texts for relevance and eligibility, a total of 52 studies were included in the final synthesis. Of these, 17 primary empirical studies are summarized in Table 1, representing observational, experimental, and randomized controlled designs. The remaining studies were used to provide contextual or mechanistic insights relevant to cognitive performance, nutrition, lifestyle behaviors, and supplementation in esports.

Given the developing state of esports research, this review aimed to provide a comprehensive and critical synthesis of current evidence rather than a formal systematic or meta-analytic evaluation.

To synthesize the findings on dietary supplements, a five-tier evidence grading system was developed for this review. Each intervention was assigned a Level of Evidence (High, Moderate, Emerging, Low/Extrapolated, or Low/Preliminary) according to three criteria: (1) the quantity and methodological quality of esports-specific human studies; (2) the consistency of findings across related cognitive-performance domains; and (3) the strength of the underlying biological mechanism.

Evidence was classified as High when at least two randomized controlled trials conducted in esports or gaming populations demonstrated consistent performance benefits. Moderate evidence reflected the presence of at least one controlled study in esports populations supported by consistent findings from related cognitive-performance literature. Emerging evidence indicated preliminary esports-specific studies or a strong mechanistic rationale but limited empirical replication. Low/Extrapolated evidence referred to compounds supported primarily by studies conducted in non-esports populations (e.g., general cognitive or sports-performance research). Low/Preliminary evidence described compounds supported only by small pilot studies or inconsistent findings with substantial methodological limitations.

A summary of the operational criteria used to assign evidence levels is presented in Table 2, and this framework is applied to the dietary supplement summary table presented in Section 5 to assist readers and practitioners in interpreting the relative strength of the available evidence.

## 3. Psychophysical Health and Lifestyle Behaviors

Research on esports athletes increasingly highlights the close relationship between lifestyle habits, physical health, and psychological well-being. A large international cross-sectional survey conducted by Trotter et al. (2020) [21] involving 1772 esports players from 65 countries examined associations between gaming involvement and health behaviors, including physical activity, BMI, smoking, and alcohol consumption. The authors reported that higher competitive engagement was not necessarily associated with poorer health outcomes and, in some cases, was linked to higher levels of physical activity and better perceived general health [21]. Despite long training hours, top-level competitors were found to engage in more frequent exercise and report better overall health compared to their lower-ranked peers, though only a minority (19.7%) met the World Health Organization’s guidelines for physical activity [21,27]. At the same time, the prevalence of overweight and obesity, alongside a substantial proportion of players classified as normal weight, suggests the need for structured exercise interventions to support both health and long-term performance capacity [21,25,27].

From a physiological perspective, esports present a dual challenge: competitive gameplay elicits acute physiological stress, while the broader training context is characterized by prolonged sedentary exposure. In controlled experiments, elite players demonstrated significant increases in energy expenditure, oxygen consumption, and heart rate during gameplay, comparable to moderate-intensity physical activity [22,28,29]. However, prolonged sedentary postures remain a major concern. Evidence suggests that esports athletes are prone to fatigue, musculoskeletal discomfort, headaches, and visual strain, particularly after extended sessions, underscoring the need to balance performance demands with preventive health strategies related to posture, movement, and recovery to maintain training consistency and competitive readiness [11,21,25].

Beyond the physical domain, psychological and behavioral factors act as important performance modifiers. Anxiety, depression, poor sleep quality, and unhealthy dietary patterns are commonly reported in esports populations. In a cross-sectional study of 292 esports football players, Pereira et al. (2021) reported that nearly one-quarter of participants experienced depressive symptoms, while almost half reported sleep disturbances [24]. Coping strategies were shown to significantly influence these outcomes: dysfunctional coping was associated with higher alcohol consumption, sleep disorders, and increased anxiety, whereas adaptive strategies served a protective role [24]. The role of stress was further emphasized in physiological studies, which documented cardiovascular and metabolic responses during competition, reflecting the high psychological intensity of esports and its potential impact on performance stability [5,22,30]. Similar stress-recovery dynamics have been described in traditional sports through overtraining syndrome, which arises when training demands exceed recovery capacity. Evidence from amateur football players indicates that psychological stress, nutrition, and training load interact in the development of overtraining-related symptoms, highlighting potential parallels with the pressures experienced by esports athletes [31].

Within this context, targeted interventions aimed at cognitive and psychophysical resilience may offer meaningful benefits. Lachowicz et al. (2024) [23] investigated the effects of a short-term virtual reality (VR) training program on reaction time, motor speed, visuomotor coordination, and execution accuracy in amateur esports players. A total of 66 participants were randomly allocated to an intervention group that completed eight consecutive daily VR training sessions and a control group with no intervention. The results demonstrated significant improvements in visuomotor coordination and execution accuracy, with improvements in coordination persisting at a 31-day follow-up, suggesting that brief VR-based training interventions may positively influence neuromotor performance relevant to esports competition [23]. Other cognitive training approaches, such as neurofeedback, have also been explored in performance and attention-regulation contexts, although empirical evidence specifically in esports populations remains limited.

Finally, lifestyle behaviors extend to sensory health. In a survey-based study of 488 participants, Diviani et al. (2025) [26] investigated listening habits, awareness, and attitudes toward the risks of hearing loss associated with exposure to loud in-game audio. The findings showed that a substantial proportion of players reported symptoms such as tinnitus and ear pressure after gaming sessions, while nearly one-quarter reported using high or very high audio volume levels. Despite relatively high awareness of potential hearing risks, only a minority of players reported adopting protective behaviors such as taking regular breaks or adjusting volume levels [26]. Overall, current evidence suggests that optimizing lifestyle behaviors and preventive health strategies may be critical for maintaining both performance and long-term well-being in esports athletes.

## 4. Nutrition and Cognitive and Physiological Function

### 4.1. Macronutrients and Micronutrients

Nutrition is widely recognized as a cornerstone of athletic performance, yet in esports, its role remains comparatively underexplored. Although awareness of its importance is increasing, current evidence suggests that many esports athletes adopt dietary patterns that fall short of supporting their cognitive and physical demands [6,7,9,10,13,32]. It is important to note that much of the available evidence is derived from cross-sectional or self-reported studies and short-term interventions. Readers should therefore interpret associations cautiously while considering the need for more controlled, esports-specific research. Several studies have shown that inadequate nutrition, whether through meal skipping, insufficient daily caloric intake, or reliance on processed foods with limited consumption of fruits and vegetables, may undermine both performance and long-term health outcomes [6,7,9,10,13,32].

From a physiological perspective, carbohydrates represent the primary energy substrate for the brain and central nervous system, and insufficient carbohydrate availability or poorly timed intake may contribute to mental fatigue, impaired attention, and slower reaction speed during extended periods of gameplay [6,7]. While carbohydrate strategies are well established in traditional sports for delaying physical fatigue, their role in esports is likely related to maintaining glucose stability during prolonged cognitive effort. However, the application of these principles to competitive gaming remains largely extrapolated from broader cognitive and sports nutrition literature.

Protein intake also plays a dual role in esports. Beyond its established importance for muscle maintenance and recovery, sufficient protein consumption has been associated with improved cognitive performance, possibly through effects on neurotransmitter synthesis and satiety regulation [6,7]. In contrast, dietary fat quality appears more relevant than total fat intake. Diets high in saturated fats and low in unsaturated fatty acids are associated with poorer cardiometabolic profiles and may negatively influence cognitive flexibility, whereas diets rich in unsaturated fats, particularly omega-3 fatty acids, support brain cell structure and neurovascular function [6,7,15,33].

At the same time, research points to a positive association between micronutrients and enhanced cognitive performance in esports. In a cross-sectional study of 119 esports athletes, Goulart et al. (2023) [7] identified that higher intakes of polyunsaturated fatty acids (PUFA), selenium, zinc, and B-vitamins were significantly correlated with superior cognitive performance scores. Conversely, this same study highlighted that poor sleep quality was a primary negative predictor of cognitive outcomes in this population [7]. These findings are in line with broader evidence showing the role of micronutrients and polyunsaturated fatty acids in supporting executive function, reaction time, and neural resilience [33]. Moreover, antioxidant-rich foods, such as vegetables, were linked to greater attentional performance, likely through reductions in oxidative stress and inflammation [6,7,9,10,13,32].

### 4.2. Dietary Patterns

Rather than isolated nutrients, overall dietary patterns appear to influence both cognitive and physiological health. Observational studies from Portugal, Brazil, Poland, and Germany report dietary behaviors among esports athletes characterized by frequent consumption of fast foods, sugary beverages, and energy drinks, alongside low intake of fruits, vegetables, and fish [9,10,13]. In several of these studies, adherence to Mediterranean-style dietary patterns was assessed as an index of diet quality and was generally reported to be low.

For example, Ribeiro et al. (2023) examined 579 esports players from Portugal and Brazil and reported that 53.7% demonstrated low adherence to the Mediterranean diet, while 84.5% failed to meet recommended fruit and vegetable intake [13]. Participants also reported frequent consumption of fast food, processed meats, sweets, soft drinks, and caffeinated beverages. Interestingly, supplement use was positively associated with greater adherence to Mediterranean-style dietary patterns, suggesting that players with higher diet quality may also demonstrate greater engagement with performance-related health behaviors [13]. Similarly, a three-year cohort study of 233 Polish esports players identified nine dietary patterns, eight of which were classified as unhealthy, reflecting frequent intake of sugar-sweetened beverages, snacks, and fried foods, as well as irregular meal patterns [10]. Cross-sectional research in German esports players further reported elevated consumption of red meat, fast food, and energy drinks compared with the general population, alongside sedentary behavior averaging 7.7 h per day [9]. Collectively, these findings suggest that dietary habits among esports athletes often resemble a Westernized dietary model characterized by high intakes of sugar, sodium, and saturated fat and low intakes of fiber and micronutrients. Such dietary patterns have been associated in broader populations with increased cardiometabolic risk and impaired cognitive function.

In contrast, Mediterranean-style dietary patterns, characterized by high consumption of fruits, vegetables, whole grains, legumes, fish, olive oil, and nuts, are consistently associated with improved cardiometabolic health and cognitive outcomes, with many of these benefits attributed to the bioactive compounds and functional properties of the foods that compose this dietary pattern, suggesting potential relevance for esports populations [13]. Supporting this perspective, Alkan et al. (2025) reported that university esports players demonstrated poorer overall dietary quality and less favorable lifestyle profiles compared with non-esports peers, reinforcing concerns about the long-term health implications of prevailing dietary patterns not fully aligned with Mediterranean principles [32]. More broadly, evidence suggests that esports athletes frequently adopt irregular eating patterns and do not meet total energy intake requirements, raising concerns about their capacity to sustain performance across repeated training and competitive schedules [6,7,9,10,13,32].

### 4.3. Mental Health and Nutrition

Importantly, the consequences of these patterns extend beyond physical health. Soffner et al. (2023) specifically noted that despite reporting positive self-rated health, many players displayed elevated BMI, physical inactivity, and reliance on convenience foods and energy drinks, with implications for both mental well-being and performance [9]. Furthermore, Arslan et al. (2024) [14] reported that more than one in five esports athletes displayed signs of food addiction, and over 13% reported night eating syndrome. Risk factors included meal skipping, longer gaming hours, and poor sleep quality, behaviors that are closely tied to esports’ unique demands and schedules [14]. In combination, these findings underscore that nutrition is not only a matter of fueling performance but also a matter of protecting mental health and preventing maladaptive coping mechanisms [9,14].

From a biological standpoint, emerging evidence highlights the relevance of the gut–brain axis. Kulecka et al. (2023) [15] performed a comparative analysis between 109 esports players and traditional endurance athletes, finding that the esports cohort exhibited significantly reduced microbial diversity and lower levels of short-chain fatty acids (SCFA). These findings suggest a unique biological profile in gamers that may be linked to their reported lower intakes of energy and protein compared to traditional athletes [15]. Such differences could have important implications, given the established gut–brain axis and its proposed influence on cognition, mood, and inflammation, although current evidence in esports remains largely associative. These insights point toward nutrition not only as behavioral input but also as a biological modulator of performance and resilience. Beyond cognitive effects, reduced microbial diversity and lower short-chain fatty acid production have been associated with impaired metabolic efficiency and inflammatory control in other athletic and clinical populations, and may therefore represent plausible mechanisms influencing fatigue tolerance and recovery capacity in esports athletes [15].

## 5. Dietary Supplements

Dietary supplements are increasingly discussed within esports communities, particularly in the form of ergogenic or cognitive-enhancing products. In contrast to traditional sports, where supplementation often targets strength or endurance, esports-related supplementation strategies primarily aim to enhance cognitive performance, reaction speed, vigilance, and resistance to mental fatigue [34,35]. Among the compounds investigated to date, caffeine represents the most consistently substantiated supplement, supported by multiple randomized controlled trials in gaming contexts. Amino acid–based formulations show promising but still preliminary evidence, whereas creatine and plant-derived bioactives remain emerging areas of investigation. The following section synthesizes the evidence according to its relative strength, and a summary of the proposed mechanisms and corresponding level of evidence for each supplement is presented in Table 3 (see Table 2 for operational criteria).

The supplements discussed in this section were selected based on their relevance to the cognitive and physiological demands of esports performance. Selection was guided by three considerations: (1) evidence linking the compound to cognitive functions relevant to gaming performance (e.g., attention, reaction time, executive function, or resistance to mental fatigue); (2) plausible mechanisms influencing neurophysiological processes such as cerebral energy metabolism, neurotransmitter activity, neurovascular function, or visual processing; and (3) the availability of human studies examining cognitive performance outcomes, particularly in gaming or comparable cognitively demanding task environments. Nutrients primarily associated with long-term neurophysiological support, such as long-chain polyunsaturated fatty acids (PUFAs), are acknowledged for their importance in brain health but are discussed within the broader nutrition and dietary patterns sections due to their predominantly chronic physiological effects.

### 5.1. Caffeine

Among all supplements, caffeine remains the most consistently studied and has been shown to enhance multiple domains of cognitive function, including reaction time, sustained attention, and alertness, largely through antagonism of adenosine receptors and modulation of central nervous system arousal [18,19]. By blocking adenosine’s inhibitory signaling, caffeine facilitates the increased release of excitatory neurotransmitters, primarily dopamine and norepinephrine, which lowers the threshold for neuronal firing and enhances sustained focus during repetitive tasks. These effects have also been demonstrated in gaming and esports contexts. Recent randomized controlled trials have quantified these effects specifically in competitive gaming. For instance, Rogers et al. (2024) found that dosages of 1 mg/kg and 3 mg/kg improved reaction time and aiming performance in 24 FPS players [18]. Similarly, Wu et al. (2024) demonstrated that 3 mg/kg of caffeine significantly enhanced shooting accuracy and kill ratios in a cohort of 9 male esports athletes [19]. However, studies also highlight potential downsides, including increased anxiety, sleep disturbances, and cardiovascular strain, particularly when consumed in high doses or late in the day [18,19].

In practical esports environments, caffeine is frequently consumed through commercial energy drinks rather than isolated supplements. These formulations typically combine caffeine with additional ingredients such as taurine, B-vitamins, guarana, and various herbal extracts. Surveys of esports athletes and gaming populations report frequent consumption of energy drinks during both training and competition sessions, often motivated by the desire to maintain alertness and delay fatigue [9,10,13]. While the ergogenic effects observed are likely driven primarily by caffeine itself, the high sugar content and cumulative stimulant load of many commercial formulations may increase the risk of excessive caffeine intake, potential fluctuations in glycemic response, sleep disruption, and cardiovascular strain when consumed repeatedly during extended gaming sessions [9,10,13,18,19]. Consequently, moderation and careful monitoring of total daily caffeine intake remain important considerations for esports athletes and practitioners.

### 5.2. Amino Acids

Amino acid supplementation has shown promising but preliminary results. Arginine, a nitric oxide precursor, has been investigated for its potential to enhance cerebral blood flow and cognitive performance. In some studies, arginine has been administered in combination with inositol, a vitamin-like compound that functions as a secondary messenger in intracellular signaling pathways influencing neurotransmitter receptor sensitivity [16,17,36]. Tartar et al. (2019) conducted a double-blind, placebo-controlled trial with 60 participants, finding significant improvements in executive function and mood following acute ingestion [16]. This was further supported by Sowinski et al. (2021), who utilized a crossover design with 26 esports athletes to demonstrate that the same dosage improved inhibitory control and working memory, despite no significant changes in actual in-game performance metrics [17]. While the present evidence focuses primarily on supplementation protocols involving arginine and related compounds, adequate dietary protein remains a prerequisite for optimal neurotransmitter synthesis and may influence responsiveness to supplementation [6]. These findings suggest that protein intake and amino acid availability may support the cognitive demands of esports, though current evidence remains limited to short interventions and small cohorts [6,16,17,36].

### 5.3. Creatine

Creatine, traditionally recognized for its role in high-intensity physical performance, has recently attracted interest in its potential cognitive benefits, particularly in tasks requiring rapid information processing and executive control. Mechanistically, creatine functions as a rapid phosphate donor in the phosphocreatine system, helping to buffer cellular energy demands and support ATP resynthesis in tissues with high metabolic activity, including the brain. Preliminary evidence in esports populations suggests that short-term creatine supplementation may improve reaction time, attention, and in-game performance metrics [37]. Beyond esports-specific research, a broader body of literature in healthy adults suggests that creatine supplementation may support aspects of cognitive performance, particularly under conditions of high mental demand, sleep deprivation, or fatigue. For example, Nejad et al. (2024) demonstrated that a single dose of creatine improved cognitive performance and increased cerebral high-energy phosphate availability during sleep deprivation [38]. These findings are biologically plausible, given creatine’s established role in cerebral energy metabolism and ATP resynthesis, which may be particularly relevant during prolonged cognitively demanding gameplay [39]. However, larger randomized controlled trials are required to confirm efficacy and establish optimal dosing strategies within esports settings.

### 5.4. Herbs and Plant-Derived Bioactives

Interest has also expanded into plant-derived compounds and novel supplements. Many of these compounds exert their effects through antioxidant activity, modulation of cerebral blood flow, or neuroprotective pathways that may support cognitive performance during prolonged mental effort. *Mangifera indica* extract has been associated with improved working memory and reduced mental fatigue, while lutein, a carotenoid with neuroprotective properties, has been linked to better visual processing speed and accuracy [20,40]. Similarly, beetroot-derived nitrates, widely studied in traditional sports, may hold relevance for esports by enhancing cerebral blood flow, endothelial function, and oxygen delivery to neural tissues, mechanisms that could support sustained attention and cognitive endurance during prolonged gameplay [41,42]. However, evidence supporting nitrate supplementation in esports remains indirect and is largely extrapolated from studies in endurance exercise and general cognitive performance. Polyphenol-rich compounds, such as cocoa flavanols and berry extracts, have demonstrated short-term benefits for attention and executive function in cognitively demanding tasks, potentially offering further support against mental fatigue [43,44]. Despite these promising mechanisms, plant-derived supplements may present additional considerations related to product standardization and contamination risk. Variability in bioactive content and the potential presence of undeclared stimulant compounds have been reported in certain commercial products, raising concerns regarding quality control and regulatory oversight. In competitive settings, such inconsistencies may pose both health risks and inadvertent doping concerns, underscoring the importance of third-party testing and cautious integration of plant-derived supplements into esports performance strategies [20,40,41,42,43,44,45].

## 6. Discussion

The findings of this review emphasize the central role of nutrition, supplementation, and lifestyle behaviors in shaping both performance and health among esports athletes. A consistent theme across studies is the contrast between the physiological and cognitive demands of competitive gaming and the often suboptimal habits adopted by players. While elite competitors tend to report higher engagement in physical activity and greater awareness of health risks, the broader esports population frequently displays sedentary habits, disrupted sleep, and reliance on processed foods and energy drinks [9,10,13,21,32]. This duality highlights the paradox of esports: where performance optimization is often pursued through acute strategies such as caffeine or extended training, while foundational practices such as diet quality, sleep, and recovery receive less systematic attention. Multiple factors may contribute to this tendency, including a competitive culture that equates high training volume with skill mastery, limited access to multidisciplinary health support outside elite tiers, and the immediate cognitive feedback provided by stimulants compared with the delayed physiological benefits of nutritional and lifestyle interventions.

Although direct links between nutrition, supplementation, and lifestyle behaviors and competitive outcomes (e.g., tournament rankings or win rates) remain limited in the current literature, several studies suggest meaningful associations with performance-related cognitive and physiological markers. Variables such as reaction time, attentional control, decision-making speed, and resistance to mental fatigue represent critical determinants of in-game performance and are frequently used as proxies for gaming success in esports research. For example, higher competitive rankings have been associated with superior dietary quality and greater engagement in physical activity among esports players [7,21], while acute interventions with caffeine and certain amino acids have been linked to improvements in aiming accuracy, reaction speed, and inhibitory control in experimental gaming tasks [16,17,18,19,36]. Nutritional adequacy, targeted supplementation strategies, and supportive lifestyle practices may therefore influence competitive outcomes indirectly by sustaining the cognitive endurance and neuromotor precision required during high-level gameplay.

Nutritionally, the body of evidence indicates that both macronutrient and micronutrient intake play a meaningful role in shaping cognitive and physiological outcomes in esports athletes. Carbohydrates serve as the primary substrate for brain energy metabolism and appear important for maintaining attention and reaction speed during prolonged gameplay, while adequate protein intake supports neurotransmitter synthesis, satiety, and post-session recovery [46,47]. Fat quality, rather than total fat intake, also appears relevant, with unsaturated fats, particularly omega-3 fatty acids, linked to more favorable cognitive and cardiometabolic profiles [7,13,15,33]. Rather than any single nutrient being decisive, these findings collectively suggest that inadequate energy availability and poor diet quality may compromise fatigue resistance, recovery capacity, and performance consistency across training and competition. Given the cognitive demands of esports, personalized nutrition strategies may be warranted. Timing of carbohydrate intake (e.g., differentiation between competition and training days), inter-individual variability in sleep chronotype and stress reactivity, and cultural or geographic dietary constraints should be considered when developing applied nutrition frameworks for esports athletes.

Beyond nutrients and energy requirements, dietary patterns and underlying biological mechanisms provide additional insight. Mediterranean-style eating patterns, characterized by high intakes of fruits, vegetables, whole grains, fish, olive oil, and nuts, are consistently linked to better cardiometabolic profiles and cognitive outcomes and may represent a practical nutritional framework for esports populations [13,48]. In parallel, emerging research on the gut–brain axis suggests that reduced microbial diversity and lower short-chain fatty acid production observed in esports players may influence cognition, mood, inflammation, and fatigue tolerance, although evidence in esports remains largely associative [15,49]. Evidence relevance may also extend beyond short-term performance and toward long-term career sustainability. Given the sedentary nature of esports training and the early age at which many players enter competitive circuits, suboptimal dietary patterns and insufficient total energy intake increase the risk of non-communicable diseases, including obesity, metabolic syndrome, and cardiovascular dysfunction, which can impair both health and sustained competitive capacity [32].

Supplementation research offers cautious optimism but remains constrained. Although caffeine demonstrates the most reliable short-term performance benefits in esports contexts, its habitual use warrants careful consideration. Individual sensitivity, sleep quality, competition timing, and long-term health implications should inform its integration into structured training routines [18,19]. Creatine monohydrate may represent a promising adjunct given its role in cerebral energy metabolism and preliminary findings suggesting improvements in reaction time and executive function [37,38,39]. Amino acids such as arginine and inositol show preliminary benefits for attention and processing efficiency, while plant-derived, nitrate-rich compounds and polyphenol-rich sources represent emerging areas of interest [16,17,20,36,40,41,42,43,44]. Overall, supplements appear most effective when integrated within optimized nutrition, sleep, and structured training frameworks rather than used as standalone performance strategies. Interpretation of current findings is further complicated by insufficient control of confounding lifestyle variables, including diet quality, sleep, stress load, and physical activity.

Lifestyle factors remain critical for performance sustainability. Sleep quality, stress management, and coping strategies are consistently identified as determinants of both psychological resilience and competitive success [12]. Promising interventions, such as structured physical conditioning, cognitive training, and health education programs, have shown effectiveness in reducing fatigue and enhancing focus, yet their uptake in professional practice remains limited [50,51].

Beyond traditional approaches, digital health technologies and artificial intelligence (AI) tools offer significant potential for personalized monitoring of diet, sleep, stress, and performance metrics in esports [52]. AI-driven platforms could facilitate adaptive interventions by integrating wearable sensor data and nutritional tracking systems [53]. However, validation in esports populations remains limited, and ethical considerations, including data privacy, algorithmic bias, and over-reliance on automated decision-making, require careful attention. AI should therefore be positioned as a decision-support tool designed to augment, rather than replace, professional oversight by credentialed practitioners.

Demographic characteristics such as age and biological sex may influence nutrition-related behaviors and physiological responses in esports populations. Most available studies predominantly involve young adult male participants, reflecting the current demographic composition of competitive gaming communities. Age-related factors may also be relevant, as competitive gaming performance has been shown to peak in early adulthood, with gradual declines in motor-response speed reported thereafter [54]. These changes may influence training demands, recovery needs, and lifestyle behaviors across different career stages. Similarly, biological sex influences factors such as body composition, hormonal regulation, and micronutrient requirements (e.g., iron status, which is critical for oxygen transport and cognitive focus) in traditional sports populations, which may also have relevance for esports athletes. Hormonal fluctuations across the menstrual cycle, for example, can influence metabolic rate, thermoregulation, and perceived fatigue, although esports-specific evidence remains limited [55]. Because female players remain substantially underrepresented in current research, the ability to draw sex-specific conclusions is currently restricted. Future studies should therefore include more diverse samples to better understand how demographic factors interact with nutrition, lifestyle behaviors, and performance outcomes in esports. Collectively, the available evidence suggests that nutrition, supplementation, and lifestyle behaviors operate as interconnected determinants of performance and health in esports. Performance optimization in this context appears to depend less on isolated interventions and more on the integration of dietary adequacy, recovery practices, structured training, and individualized support within a coherent performance framework.

## 7. Limitations and Future Research Directions

A further limitation of the current evidence base is the reliance on data extrapolated from traditional sports nutrition and broader cognitive-performance research. While the underlying biological mechanisms involved, such as cerebral energy metabolism, neurovascular coupling, and neurotransmitter modulation, are broadly applicable to human performance, the ecological validity of these findings within the unique competitive environment of esports remains to be fully established. Esports athletes experience distinct demands, including prolonged screen exposure, high-frequency motor responses, psychological pressure, and irregular competition schedules, which may modify how nutritional and supplemental strategies influence performance and health outcomes. Consequently, further esports-specific experimental research is required to confirm the applicability of these interventions in competitive gaming contexts.

Beyond the challenges associated with extrapolating findings from traditional sports and cognitive-performance research, the current body of evidence in esports nutrition and supplementation is also characterized by several methodological constraints. Many studies rely on cross-sectional designs, self-reported dietary and lifestyle measures, small sample sizes, and short intervention periods, limiting the ability to establish causal relationships and increasing the risk of bias. Specifically, it remains unclear whether healthy dietary patterns directly enhance cognitive performance or whether athletes with higher baseline cognitive abilities naturally adopt healthier habits. Moreover, confounding factors, such as sleep quality, stress load, physical activity, and training volume, are inconsistently controlled, complicating interpretation of observed associations.

To strengthen the evidence base, future research must prioritize longitudinal, well-powered intervention trials conducted in ecologically valid esports environments. Studies should assess both acute and chronic effects of nutrition and supplementation on cognitive performance, recovery, metabolic health, and markers of career longevity. Incorporating objective biomarkers, physiological measures, and standardized performance metrics will clarify underlying mechanisms of action and improve reproducibility. Greater attention should also be directed to personalization, considering timing strategies for training versus competition and cultural or geographic dietary constraints, while integrating nutritional interventions with holistic approaches to athlete health.

Beyond methodology, several practical and regulatory considerations regarding supplements remain underaddressed in esports. Unlike traditional sports, esports currently lack standardized supplement guidelines, clear anti-doping frameworks, and consistent oversight. The variability in supplement quality and the risk of contamination with undeclared substances represent major concerns, particularly given the widespread use of energy drinks, nootropic blends, and commercially marketed performance products. Strengthening quality assurance practices, promoting third-party testing, and clarifying governance structures are essential steps toward minimizing health risks and inadvertent rule violations within competitive settings.

Collectively, advancing the field will require greater methodological rigor, interdisciplinary collaboration, and the development of esports-specific guidelines that translate emerging evidence into safe, practical, and sustainable applications.

## 8. Conclusions

This narrative review highlights the complex and multifaceted demands placed on esports athletes and underscores the central role of nutrition, supplementation, and lifestyle behaviors in supporting both performance and long-term health. Although esports are frequently conceptualized as predominantly cognitive activities, the evidence demonstrates that competitive gaming imposes a unique physiological contradiction: the high metabolic and cognitive load of elite competition superimposed on a largely sedentary physical profile.

While inadequate dietary patterns and suboptimal lifestyle behaviors remain prevalent, this review suggests that esports athletes require a shift from viewing health as a secondary consideration to treating it as the primary infrastructure for cognitive precision. Within this framework, interventions such as targeted supplementation or emerging performance technologies are best understood not as standalone solutions, but as situational tools that depend entirely on the stability of foundational health behaviors. The professionalization of the field therefore necessitates a transition from reactive “performance fixes” toward proactive, multidisciplinary support structures that prioritize baseline screening, metabolic individuality, and circadian timing.

As esports continues to mature into a rigorous professional discipline, its long-term success will depend on the adoption of structured, health-centered support systems that align competitive ambition with sustainable well-being. Embedding personalized nutrition, lifestyle management, and evidence-based supplementation within professional practice, complemented by disciplined training, structured recovery, and consistent performance development, creates a holistic framework that strengthens physiological resilience, optimizes cognitive performance, and promotes career longevity. By moving beyond a “game-only” focus, this approach positions health as a competitive advantage in the modern esports era.

## 9. Key Takeaways for Practitioners

- Emphasize nutritional foundations before supplementation.

Adequate total energy intake, macronutrient balance, and micronutrient sufficiency could potentially form the basis of performance support. Strategic meal planning should reflect training load and competition schedules (e.g., training vs. rest days, match days vs. off days) to sustain cognitive endurance, recovery, and consistency. Supplements might be used selectively and monitored carefully, ideally to address specific needs rather than as primary performance solutions.

- Use supplementation as a targeted, individualized tool.

Evidence supports short-term benefits of certain supplements (e.g., caffeine, selected amino acids, plant-derived compounds), but responses vary widely. Practitioners should individualize dosing and timing, consider sleep sensitivity and stress load, and regularly reassess necessity rather than relying on habitual use.

- Recognize sleep as a key performance resource.

Sleep quality and regularity appear to play an important role in reaction time, decision-making, emotional regulation, and learning. Interventions such as sleep scheduling, light exposure management, caffeine timing, and competition-aware sleep strategies have the potential to enhance performance without increasing training volume, though further validation in professional gaming schedules is warranted.

- Consider integrating structured physical conditioning and ergonomics into daily routines.

Strength training, mobility work, and postural interventions support injury prevention, fatigue resistance, and physiological resilience. Ergonomic optimization (seating, screen height, breaks, audio exposure) may be considered part of performance preparation, not as optional health add-ons.

- Address stress management and coping skills explicitly.

Psychological stress is inherent to competitive gaming and directly influences cognitive performance. Teaching adaptive coping strategies, recovery routines, and emotional regulation skills can reduce burnout risk and improve performance stability under pressure.

- Adopt a holistic, player-centered support model.

Performance staff and coaches should look beyond a game-only focus and consider the athlete behind the screen. Behaviors outside formal training, such as nutrition, sleep, recovery, stress, and daily routines, can meaningfully shape in-game efficiency, learning capacity, and long-term development.

- Recognize that performance gains do not always come from more gameplay.

Improvements in nutrition, sleep, physical conditioning, and mental recovery can enhance focus, reaction speed, and decision-making, often more efficiently than additional hours of play.

- Embrace personalization as a core principle.

Individual variability in metabolism, chronotype, stress tolerance, and competition demands means that standardized approaches are unlikely to be optimal. Personalized, adaptable strategies represent a promising approach for sustainable esports performance support.

## Figures and Tables

**Table 1:** Summary of Empirical Studies Included in the Narrative Review.

Author	Country	Study Design	Population (N)	Key Outcome	Main Findings
Goulart et al., 2023 [7]	USA	Cross-sectional	119 esports athletes	Dietary intake & cognition	Higher intake of PUFA, selenium, zinc, B-vitamins, and vegetables was associated with better cognitive performance; poor sleep negatively associated with performance
Ribeiro et al., 2023 [13]	Portugal	Cross-sectional	579 esports players	Dietary patterns & gaming behavior	Low adherence to Mediterranean diet; high fast-food and soft drink consumption; 32% reported supplement use
Szot et al., 2023 [10]	Poland	Cohort	233 male esports athletes	Dietary patterns	Predominantly Western-type dietary patterns; low intake of brain-supportive foods
Soffner et al., 2023 [9]	Germany	Cross-sectional	817 gamers (including 210 esports athletes)	Diet & lifestyle behaviors	High sedentary time; frequent energy drink intake; suboptimal fruit and fish consumption; ~25% reported low mental wellbeing
Arslan et al., 2024 [14]	Turkey	Cross-sectional	248 esports players	Eating behaviors & sleep	Frequent meal skipping and night eating associated with longer gaming duration and poorer sleep
Kulecka et al., 2023 [15]	Poland	Cross-sectional	109 esports players	Diet & gut microbiome	Lower energy and protein intake; reduced microbial diversity and SCFA levels compared with traditional athletes
Tartar et al., 2019 [16]	USA	Randomized, double-blind, placebo-controlled trial	60 gamers	Arginine (1500 mg) + Inositol (100 mg)	Improved executive function and mood; no significant improvement in in-game performance compared with placebo
Sowinski et al., 2021 [17]	USA	Randomized, double-blind, crossover trial	26 esports athletes	Arginine (1500 mg) + Inositol (100 mg)	Improved inhibitory control and working memory; no major adverse effects reported
Rogers et al., 2024 [18]	Australia	Randomized, single-blind, crossover trial	24 FPS players	Caffeine (1 mg/kg & 3 mg/kg)	Improved reaction time, aiming performance, vigilance, and alertness; reduced perceived fatigue
Wu et al., 2024 [19]	Taiwan	Randomized, single-blind, crossover trial	9 male esports athletes	Caffeine (3 mg/kg)	Improved reaction time, shooting accuracy, kill ratio, and visual search performance
Jeyakodi et al., 2024 [20]	India	Randomized, single-blind, crossover trial	60 male gamers	*Mangifera indica* extract (300 mg daily, 7 days)	Improved processing speed, attention, and verbal learning; no significant between-group cortisol differences
Trotter et al., 2020 [21]	Australia & Sweden	Cross-sectional survey	1772 gamers	General health, BMI & physical activity	Higher in-game rank associated with greater gaming frequency and lower BMI; limited associations with perceived health, smoking, or alcohol use
Nicholson et al., 2024 [22]	Australia	Experimental laboratory study	13 elite male esports athletes	Physiological response to gameplay	Competitive gameplay increased heart rate, energy expenditure, and oxygen consumption compared with rest; no major HRV changes
Lachowicz et al., 2024 [23]	Poland	Experimental intervention study	52 amateur gamers	Visuomotor coordination & reaction	VR training improved visuomotor coordination and accuracy; some effects maintained at follow-up
Pereira et al., 2021 [24]	Portugal	Cross-sectional study (SEM analysis)	292 esports football players	Mental distress & coping strategies	25% reported depressive symptoms; 50% sleep disturbances; dysfunctional coping associated with anxiety, alcohol use, and poor dietary habits
Lam et al., 2022 [25]	China	Cross-sectional study	50 elite male esports athletes	Musculoskeletal pain & fatigue	High prevalence of neck pain, eye strain, headaches, and fatigue; most symptoms were mild
Diviani et al., 2025 [26]	International (92 countries)	Cross-sectional survey	488 gamers/esports participants	Hearing behaviors & risk awareness	High exposure to loud audio levels; frequent tinnitus and ear pressure; limited protective behaviors despite high awareness

Abbreviations: PUFA, polyunsaturated fatty acids; SCFA, short-chain fatty acids; BMI, body mass index; HRV, heart rate variability; VR, virtual reality; SEM, structural equation modeling.

**Table 2:** Operational criteria used to classify the level of evidence in this review.

Level	Operational Criteria
High	≥2 randomized controlled trials in esports/gaming populations with consistent positive findings
Moderate	≥1 controlled study in esports populations plus supportive evidence from cognitive-performance literature
Emerging	Preliminary esports studies OR strong biological plausibility with limited empirical evidence
Low/Extrapolated	Evidence derived primarily from non-esports populations
Low/Preliminary	Small pilot studies, inconsistent results, or substantial methodological limitations

Note: Evidence levels were assigned based on the quantity and methodological quality of esports-specific studies, consistency of findings across related cognitive-performance research, and the strength of the proposed biological mechanism.

**Table 3:** Summary of Dietary Supplements, Proposed Mechanisms, and Strength of Evidence for Cognitive Performance in Esports.

Supplement	Proposed Mechanism	Primary Effect(s)	Level of Evidence	Key Considerations
Caffeine	Adenosine receptor antagonism; Increase CNS arousal	Improved reaction time, sustained attention, vigilance, aiming accuracy [18,19]	High	Anxiety, sleep disruption, dose timing; critical requires individualized dosing.
Arginine	Nitric oxide pathway; possible neurovascular effects	Improved accuracy, reduced impulsivity [16,17,36]	Moderate	Small samples; short duration
Inositol	Modulation of neurotransmitter signaling	Improved attentional control [16,17,36]	Moderate	Limited replication
Creatine Monohydrate	Enhanced cerebral ATP resynthesis; energy buffering	Improved processing speed and executive function [37,38,39]	Emerging	Dose–response unclear; larger RCTs needed
*Mangifera indica* extract	Polyphenol activity; anti-fatigue mechanisms	Improved working memory, reduced mental fatigue [20]	Emerging	Product standardization variability and potential contamination risks
Lutein	Neuroprotective carotenoid; visual processing support	Improved visual processing speed and contrast sensitivity [40]	Emerging	Long-term intake required; specifically relevant for eye health
Beetroot-derived nitrates	Increase cerebral blood flow; endothelial function	Potential support for sustained attention, cerebral blood flow, oxygen efficiency [41,42]	Low/Extrapolated	Mechanism plausible; limited esports trials
Polyphenol-rich compounds (cocoa, berries)	Antioxidant; improved cerebral perfusion	Short-term improvements in attention and executive function [43,44]	Low/Preliminary	Variability in dosing and bioactive content

Note: CNS = central nervous system; ATP = adenosine triphosphate; RCTs = randomized controlled trials. Evidence levels reflect the relative strength and consistency of available esports-specific research: High = multiple RCTs in gaming/esports populations; Moderate = limited esports studies or consistent evidence in related cognitive domains; Emerging = preliminary esports data or strong biological plausibility; Low/Extrapolated = derived primarily from non-esports populations; Low/Preliminary = small pilot studies with substantial methodological limitations.

## Data Availability

No new data were created or analyzed in this study. Data sharing is not applicable to this article.
